# Impaired Spatial Firing Representations of Neurons in the Medial Entorhinal Cortex of the Epileptic Rat Using Microelectrode Arrays

**DOI:** 10.34133/research.0229

**Published:** 2023-09-15

**Authors:** Zhaojie Xu, Fan Mo, Gucheng Yang, Penghui Fan, Botao Lu, Wei Liang, Fanli Kong, Luyi Jing, Wei Xu, Juntao Liu, Mixia Wang, Yirong Wu, Xinxia Cai

**Affiliations:** ^1^ State Key Laboratory of Transducer Technology, Aerospace Information Research Institute, Chinese Academy of Sciences, Beijing 100190, China.; ^2^ University of Chinese Academy of Sciences, Beijing 100049, China.

## Abstract

Epilepsy severely impairs the cognitive behavior of patients. It remains unclear whether epilepsy-induced cognitive impairment is associated with neuronal activities in the medial entorhinal cortex (MEC), a region known for its involvement in spatial cognition. To explore this neural mechanism, we recorded the spikes and local field potentials from MEC neurons in lithium–pilocarpine-induced epileptic rats using self-designed microelectrode arrays. Through the open field test, we identified spatial cells exhibiting spatially selective firing properties and assessed their spatial representations in relation to the progression of epilepsy. Meanwhile, we analyzed theta oscillations and theta modulation in both excitatory and inhibitory neurons. Furthermore, we used a novel object recognition test to evaluate changes in spatial cognitive ability of epileptic rats. After the epilepsy modeling, the spatial tuning of various types of spatial cells had suffered a rapid and pronounced damage during the latent period (1 to 5 d). Subsequently, the firing characteristics and theta oscillations were impaired. In the chronic period (>10 d), the performance in the novel object experiment deteriorated. In conclusion, our study demonstrates the detrimental effect on spatial representations and electrophysiological properties of MEC neurons in the epileptic latency, suggesting the potential use of these changes as a “functional biomarker” for predicting cognitive impairment caused by epilepsy.

## Introduction

Spatial navigation assumes a crucial role in the survival of animals and involves complex cognitive processes such as sensing, encoding, and memory [1]. It is widely accepted that the brain forms a cognitive map to represent the surrounding environment [2]. The medial entorhinal cortex (MEC) is considered to be the central hub of the cognitive map, responsible for generating and updating dynamic representations of the animal's spatial location [3]. Increasing evidence has revealed specific coding properties of spatial information within the MEC, including grid cells [4,5], which are characterized by hexagonally arranged spike firing fields in a 2-dimensional environment, and border cells [6], which exhibit selective firing near geometric boundaries. In addition, studies have demonstrated that the theta rhythms of local field potentials (LFPs) in the MEC modulate the firing of spatial functional cells [7], resulting in the substantial theta phase locking [8]. The relationship between the neural coding properties of the MEC and spatial cognition has received considerable attention from researchers.

Epilepsy is among the most common chronic neurological disease worldwide, with a combined lifetime prevalence exceeding 0.7% [9]. Temporal lobe epilepsy (TLE), which originates from neural dysfunction in the entorhinal–hippocampal area [10], is a relatively common type of refractory partial epilepsy. Its recurrent features of spontaneous seizures severely impair patients' cognitive functions, including learning [11], memory [12,13], and language [14]. Therefore, the behavioral consequences following epilepsy have garnered increasing research attention in recent years [15,16]. MEC serves as the epileptogenic region as well as the spatial processing hub, suggesting a potential causal relationship between the epilepsy-induced spatial cognitive impairment and the functional properties of spatial cells in MEC, which warrants further investigation.

In order to investigate the impact of epilepsy on the spatial cognitive function in MEC, we utilized the microelectrode arrays (MEAs) specifically designed for MEC to detect the LFPs and neural spikes from the epileptic rat model induced by lithium–pilocarpine in the latent period (1 to 5 d) and the chronic period (14 d). Subsequently, we identified grid cells, border cells, and other nonspecific spatial cells and examined changes in their spatial tuning. Furthermore, we assessed the theta oscillations in LFPs and theta modulation in both excitatory and inhibitory neurons. Finally, we compared the spatial representations and firing properties of MEC neurons with the rats' performance in the novel object recognition test to validate the relationship between neuronal dysfunction in the MEC and cognitive deficits caused by epilepsy.

## Results

### Reduced spatial representations of MEC spatial cells in TLE rats

**Fig. 1.**
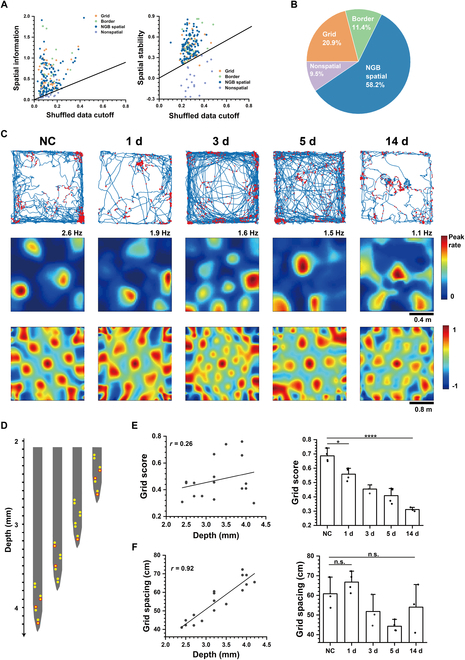
Grid cell firing patterns in relation to the duration of epilepsy and the depth in the brain. (A) Recorded MEC cells’ spatial information (left) and stabilities (right) were plotted against the 5th largest value (“cutoff”) from each cell’s shuffled data. (B) Proportion of grid, border, non-grid-border (NGB) spatial, and nonspatial cells in MEC. (C) Example of grid cells in a no-treatment control (NC) rat and 1 d, 3 d, 5 d, and 14 d after TLE modeling, shown in trajectory maps with red spike position dots (top), firing rate maps (middle), and autocorrelograms (bottom). (D) Arrangement of recording sites of the self-designed MEA according to the depth in the brain. Red circles indicate the position of detected grid cells in a rat. (E) Grid scores exhibited poor linear relationship with the depth (left) and an obvious decreasing trend with the TLE duration (right) (unpaired *t* test, *P < 0.05, ****P < 0.0001). (F) Grid spacing exhibited an obviously linear relationship to the depth of cells (left) and no significant change between NC and TLE groups (right).

MEC served as the specialized region for processing spatial information, with various types of spatial cells that exhibit distinct spatially firing patterns. Out of the total recorded MEC cells (*n* = 253), apart from 41 inhibitory neurons and 11 excitatory neurons with insufficient trajectory data, we categorized 201 excitatory cells into 42 grid cells, 23 border cells, 117 non-grid-border (NGB) spatial cells, and 19 nonspatial cells. The identification of grid cells and border cells was based on their unique spatial firing patterns (Fig. S5), as described in the Supplementary Materials. Apart from the well-defined spatial cells, we marked other cells showing high spatial firing selectivity and stabilities (refer to the Supplementary Materials for details) as NGB spatial cells. As shown in Fig. 1A, where the spike timestamps of each cell were randomly shuffled 100 times, the NGB spatial cell was identified when its spatial information and spatial stability surpassed the 95% confidence threshold derived from the shuffled data. Cells that did not meet the criteria for grid cells or border cells, and exhibited spatial information or stabilities lower than the cutoff established from the shuffled data, were classified as nonspatial cells. The percentages of grid, border, NGB spatial, and nonspatial cells are shown in Fig. 1B.

To investigate the potential impairment of spatial cognition due to epilepsy, we compared the spatial representation of grid cells between no-treatment control rats (NC) and rats with TLE induced by pilocarpine treatment in both latent period (1 d, 3 d, and 5 d) and chronic period (14 d). As shown in Fig. 1C, grid cells were visualized that the spike assembles clustered in hexagonally arranged positions. We observed that hexagonal peaks in the firing rate maps and autocorrelograms became increasingly blurry from NC to 14 d, indicating the impairment of grid selectively firing patterns. This observation was further supported by the decline in grid scores, which quantified how well the firing pattern matched the regular hexagon. In contrast, the grid patterns in the NS group did not reduce significantly over time (Fig. S6). To account for potential depth-related effects, we determined the depth of grid cells in the brain based on the arrangement of recording sites in MEA (Fig. 1D) and confirmed that cell depth did not influence the grid score obviously (Fig. 1E). In addition, we found that the grid spacing of the hexagonal pattern varied significantly across different depths. Notably, the grid spacing increased gradually from the dorsal to the ventral region of the MEC, in contrast, which was not significantly affected by TLE (Fig. 1F). These findings suggest that TLE induces a decrease in the spatial tuning of grid cells but does not interfere with the modular organization of grid patterns from the dorsal to the ventral MEC.

**Fig. 2.**
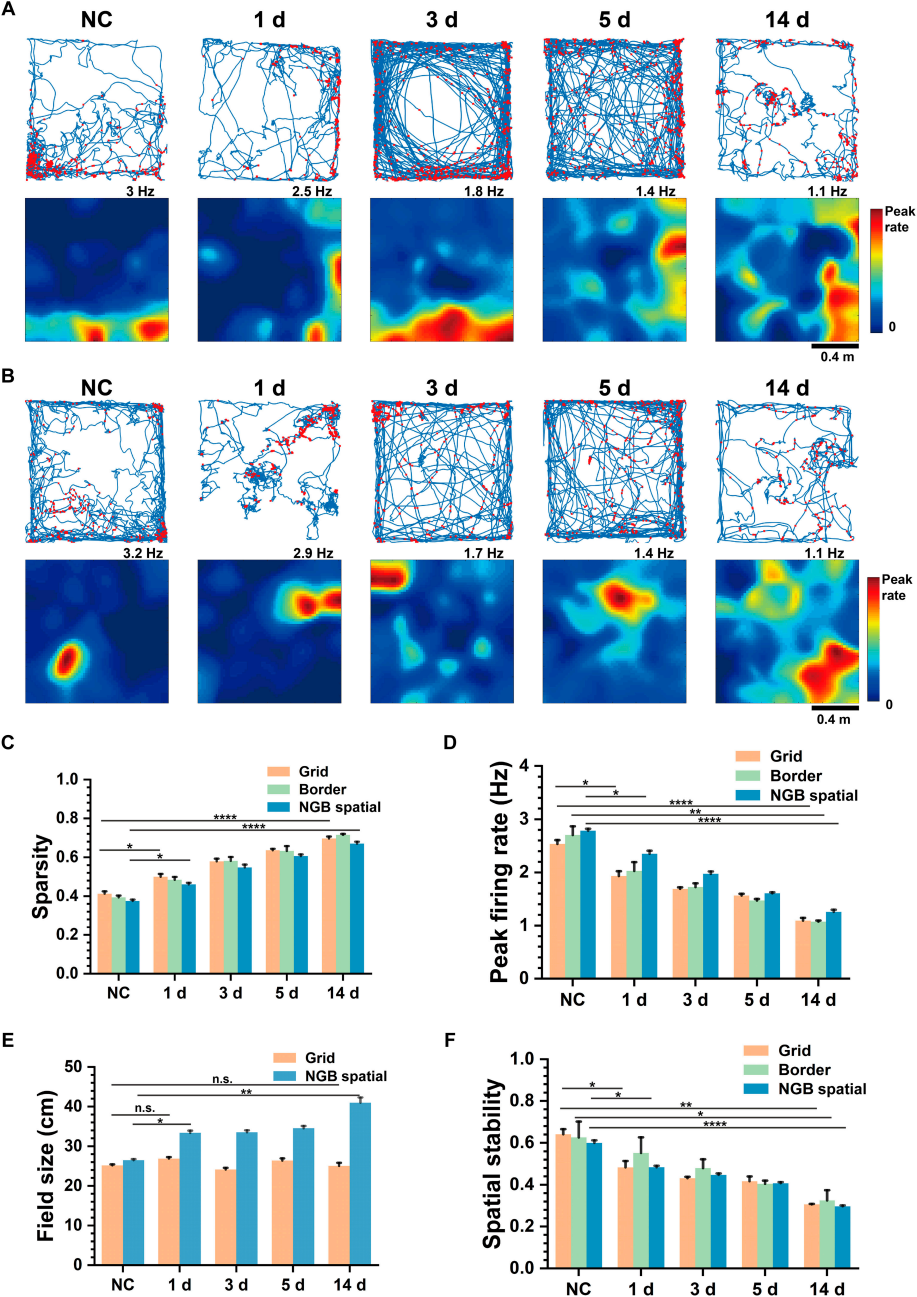
The decline of spatial representations with the duration of TLE for all types of spatial cells. (A) Increasingly dispersed spatial firing patterns of border cells from NC to TLE 14 d shown in trajectory maps with red spike position dots (top) and firing rate maps (bottom). (B) Increasingly dispersed spatial firing patterns of NGB spatial cells from NC to TLE 14 d shown in trajectory maps with red spike position dots (top) and firing rate maps (bottom). (C) The sparsity of grid, border, and NGB spatial cells gradually increases from NC to TLE 14 d (unpaired *t* test, *P < 0.05, ****P < 0.0001). (D) The peak firing rates of grid, border, and NGB spatial cells gradually reduce from NC to TLE 14 d (unpaired *t* test, *P < 0.05, **P < 0.01, ****P < 0.0001). (E) The field sizes of NGB spatial cells gradually increase from NC to TLE 14 d, but those of grid cells are comparable (unpaired *t* test, *P < 0.05, **P < 0.01). (F) The spatial stabilities of grid, border, and NGB spatial cells gradually reduce from NC to TLE 14 d (unpaired *t* test, *P < 0.05, **P < 0.01, ****P < 0.0001).

The spatial firing of border cells and NGB spatial cells showed similar dispersion trends with grid cells in the TLE group (Fig. 2A and B). To assess the spatial firing selectivity of these 3 types of spatial cells, we used the sparsity index (refer to the Supplementary Materials for details) to evaluate spike dispersion and utilized the peak firing rate as an inverse correlation measure for spatial firing selectivity (Fig. 2C and D). Statistical analysis revealed that the spatial tuning of grid cells, border cells, and NGB spatial cells in the MEC gradually weakened from NC to 14 d after TLE modeling with the similar trend. Additionally, the firing rate maps exhibited a clear trend of increasing firing field sizes. To quantify this change, we calculated field sizes defined as the mean diameters of firing fields in both grid cells and NGB spatial cells. We found that their field sizes were comparable in the NC group, but the field sizes of NGB spatial cells tended to increase more prominently with the duration of TLE compared to that of grid cells (Fig. 2E). Similar to grid spacing, the field size of grid cells enlarged from the dorsal to the ventral MEC (Fig. S8A), suggesting the difference in mechanism underlying spatial encoding between grid cells and NGB spatial cells. Furthermore, reliable reproduction of spatial firing patterns was crucial for spatial cells. To assess the repeatability of spatial firing patterns, we analyzed the spatial stabilities of all spatial cells. As depicted in Fig. 2F, the stabilities of grid cells, border cells, and NGB spatial cells exhibited a decreasing trend from the NC to TLE 14 d. Furthermore, the spatial representations of the control group did not display an evident declining trend (Fig. S7). This validates that the impaired spatial firing is attributed to TLE rather than the MEA implantation procedure.

### MEC excitatory neurons and inhibitory neurons were impaired in TLE rats

**Fig. 3.**
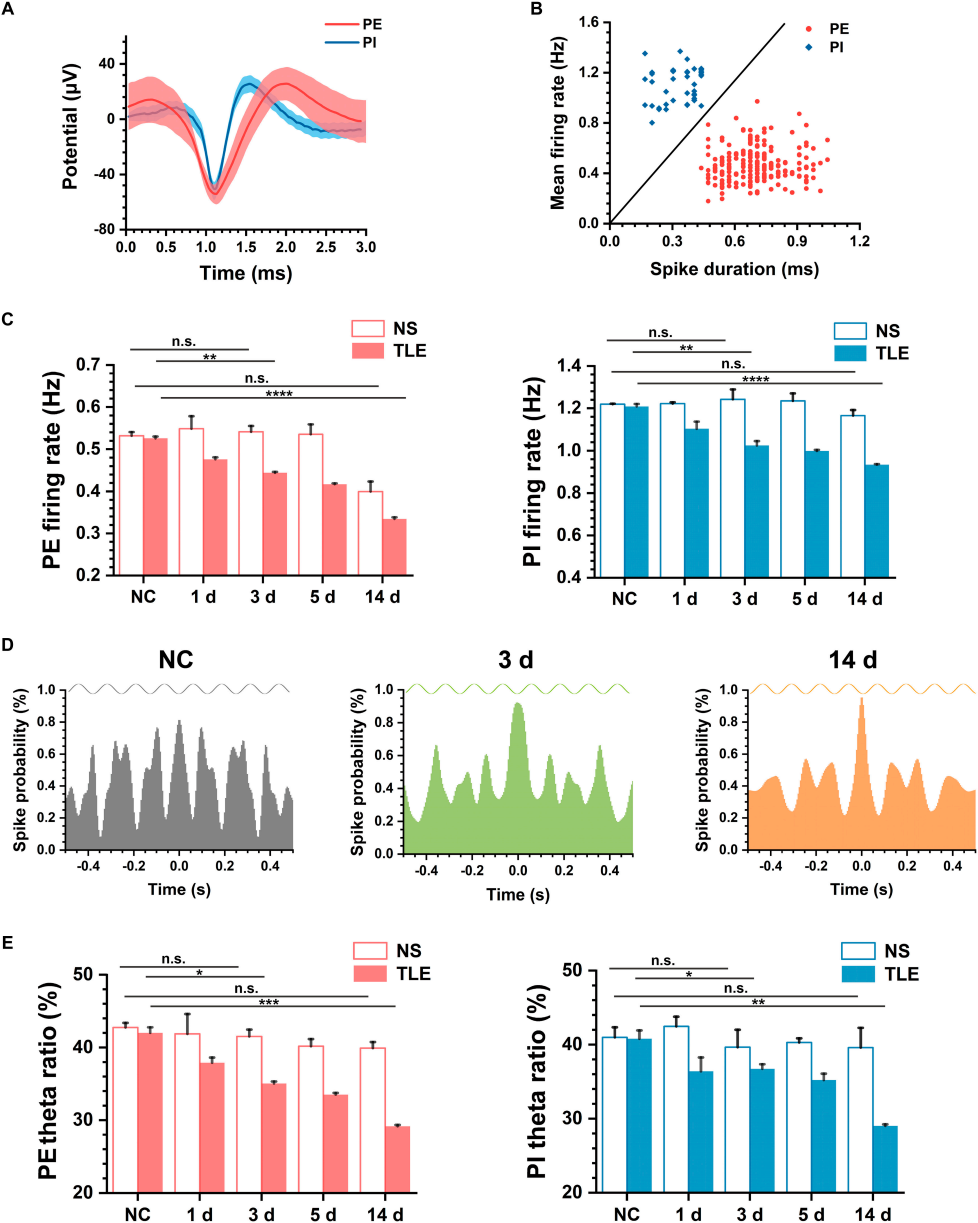
Electrophysiological characteristics of putative excitatory (PE) neurons and putative inhibitory (PI) neurons in MEC between TLE and NS. (A) Examples of mean spike waveforms of both PE and PI. (B) Mean firing rates of all recording cells versus spike duration, with PE and PI classified into 2 clusters (*n* = 253 cells). (C) Statistics of mean firing rate of recorded PE (left) and PI (right) from NC to 14 d between NS and TLE (unpaired *t* test, **P < 0.01, ****P < 0.0001). (D) Spike time autocorrelations of example recording cells in NC, TLE 3 d, and TLE 14 d, which exhibit the gradually decreased periodic peaks fit with sine wave at 8 Hz. (E) Statistics of theta ratio of spike PSD of recorded PE (left) and PI (right) from NC to 14 d between NS and TLE (unpaired *t* test, *P < 0.05, **P < 0.01, ***P < 0.001).

We further investigated whether epilepsy impacted the underlying physiology of MEC neurons. Two types of MEC neurons consisting of excitatory and inhibitory neurons were discriminated. Specifically, inhibitory neurons were characterized by higher firing rates and narrow waveform width, while excitatory neurons exhibited the opposite pattern. The waveform patterns illustrating these differences were presented in Fig. 3A. Additionally, Fig. 3B displays all 253 recorded cells, with their firing rates against the waveform width. We used *k*-means clustering to separate all the cells into 2 distinct clusters referred to as putative excitatory (PE) neurons and putative inhibitory (PI) neurons.

Subsequently, we proceeded to characterize activities of PE and PI neurons in the TLE group with pilocarpine treatment and the control group with normal saline treatment (NS). The average firing rates of each type of neuron were calculated to evaluate their instant activities. As shown in Fig. 3C, both PI and PE exhibited a gradual reduction in their average firing rates from NC to TLE 14 d. Conversely, the firing rates of these 2 cell types in the NS group showed no significant change, eliminating potential reasons such as the craniotomy injury and declined electrode performance over time. Furthermore, in comparison to the NC group, 2 weeks of TLE resulted in a 36.4% decrease in PE firing rate and a 22.7% decrease in PI firing rate, indicating that PE neurons may be more susceptible to damage caused by TLE.

We further analyzed the rhythm of spike time sequences in MEC neurons. Figure 3D illustrates the spike time autocorrelations with the peaks at 8 Hz in theta band (4 to 8 Hz), indicating the presence of theta rhythm in the spike firing. Additionally, it was evident that the theta peak decreased in 3 d after TLE modeling and further declined in 14 d. The theta ratio (see Materials and Methods) of spike firing power spectral density (PSD) in the NS group confirmed the prevalence of theta rhythms in both PE and PI neurons. In contrast, the spike theta rhythms of PE and PI neurons in the TLE group obviously decreased over time (Fig. 3E).

### Diminished MEC theta rhythm and phase locking strength with TLE

**Fig. 4.**
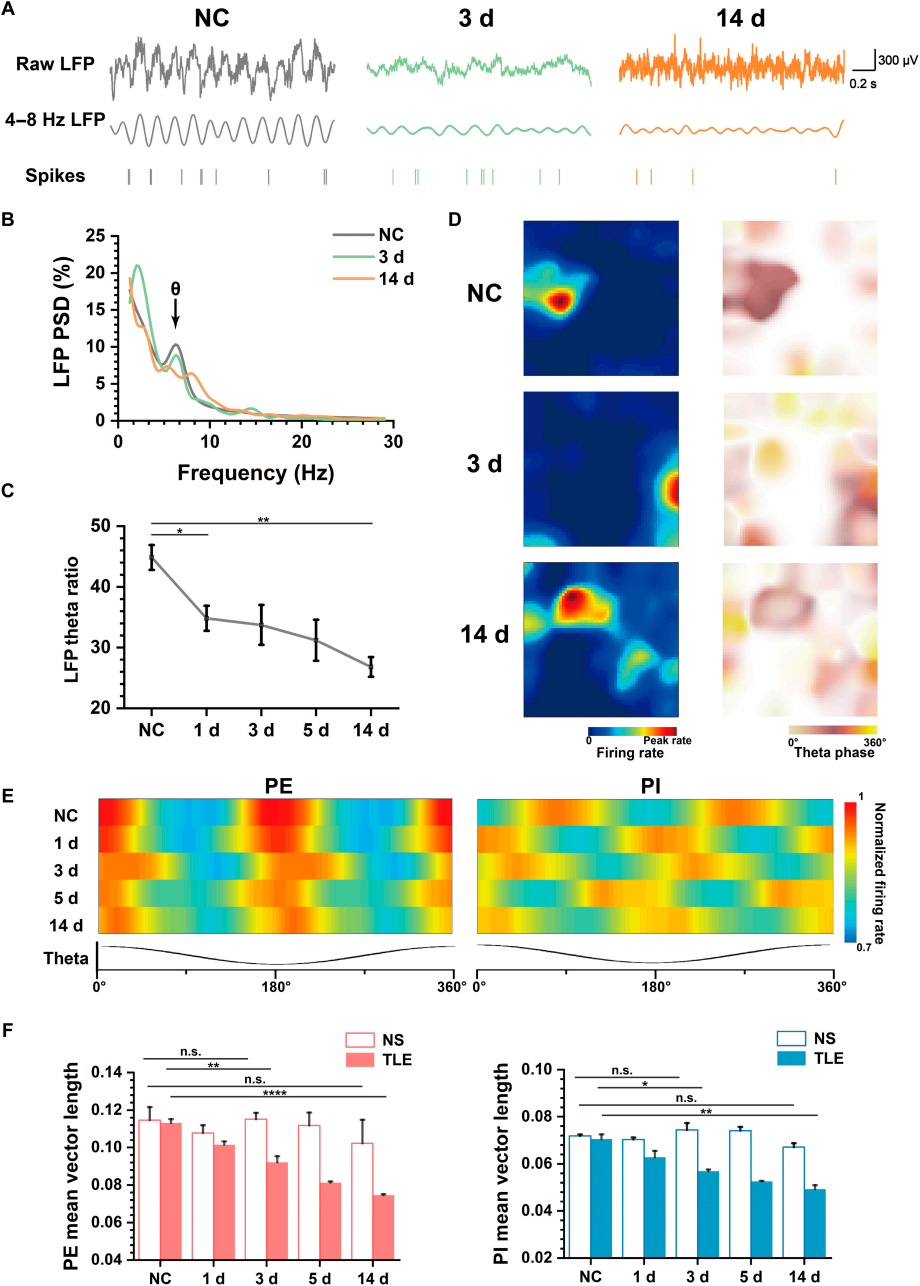
Theta oscillations and phase locking in MEC are impaired by epilepsy. (A) Examples of 3 pieces of raw LFPs (top), 4 to 8 Hz filtered LFPs in the theta band (middle), and synchronous spike raster diagram (bottom) in NC, TLE 3 d, and TLE 14 d. (B) Normalized PSD of LFPs in NC, TLE 3 d, and TLE 14 d. (C) Theta ratio of PSD of LFPs from NC to TLE 14 d (unpaired *t* test, *P < 0.05, **P < 0.01). (D) Spike firing rate maps (left) and corresponding rate maps of theta phase distributed in the open field (right) in NC, TLE 3 d, and TLE 14 d. (E) Spike rate maps of the phase distribution in the theta wave of PE (left) and PI (right) from NC to TLE 14 d. (F) Statistics of mean vector lengths of PE (left) and PI (right) from NC to 14 d between NS and TLE (unpaired *t* test, *P < 0.05, **P < 0.01, ****P < 0.0001).

Theta rhythm is a prominent characteristic of MEC LFPs, which is always accompanied with phase locking of spikes (Fig. 4A). Therefore, we analyzed whether epilepsy affected the theta rhythm and theta phase locking. Figure 4B displays the PSD of 3 representative LFP recordings from NC, TLE 3 d, and TLE 14 d, respectively. The PSD in NC exhibited a distinct peak at the theta frequency, indicating the presence of theta oscillations in MEC LFPs. Comparatively, the PSD in the TLE 3 d displayed a lower peak at the theta frequency. Furthermore, the theta peak decreased further and shifted toward higher frequencies in TLE 14 d. The reduction in theta power in LFPs was further supported by the statistical analysis of the theta ratio shown in Fig. 4C, which corresponded to the decrease in spike theta power.

Theta phase locking frequently occurred during recording sessions, with spike firings often aligning with specific phases of the theta rhythm. Figure 4D illustrates that in the firing fields, spike theta phases predominantly clustered around 180°, while phases outside the firing fields appeared more dispersed. However, in TLE 3 d and 14 d, the occurrence of nonlocked phases outside the firing fields significantly increased, which indicated the decrease in phase locking strength. As shown in Fig. 4E, 2 heat maps exhibited the normalized firing rate distributions across different theta rhythm phases of PE and PI from NC to TLE 14 d. The spikes of PE were gathered around 180° of theta phases all the time, whereas the locked phases of PI were random and the spike distributions were relatively diffuse, suggesting the weaker phase locking strength. In comparison to NC, the spike distributions of TLE rats were gradually dispersed from 1 d to 14 d. To further verify the relationship between phase-locked strength and TLE, we calculated the mean vector lengths of each cell. As depicted in Fig. 4F, the shorter mean vector lengths of PE and PI neurons in the TLE group compared to those in the NS group indicated that phase locking strength was reduced in both latent period and chronic period.

### Recognition abilities were damaged after impairment of spatial representations and electrophysiological characteristics

**Fig. 5.**
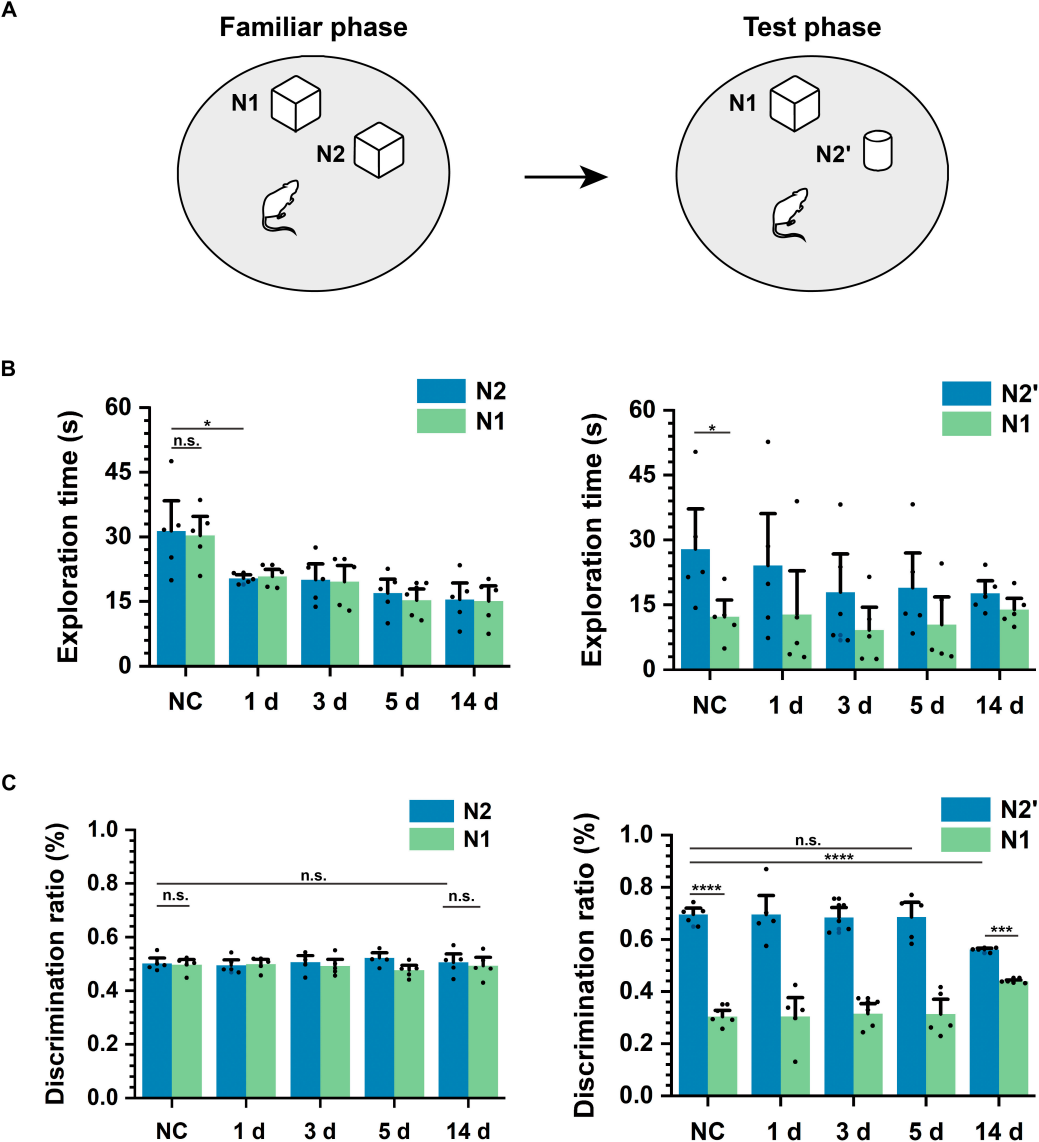
Impaired performance of TLE rats in the novel object recognition experiment. (A) Schematic diagrams of the novel object recognition experiment, which contain the familiar phase and the test phase. (B) Exploration time for 2 objects in the familiar phase (left) and the test phase (right) from NC to TLE 14 d (*n* = 5; paired *t* test, *P < 0.05, **P < 0.01). (C) Discrimination ratio for 2 objects in the familiar phase (left) and the test phase (right) from NC to TLE 14 d (*n* = 5; paired *t* test, ***P < 0.001, ****P < 0.001).

To explore the impairment in spatial cognition of rats over the duration of TLE, we analyzed the performance in the novel object recognition experiment from NC to TLE 14 d (Fig. 5A). The exploration time and discrimination ratio for each in 2 phases were counted as shown in Fig. 5 B and C. During the familiar phase, the exploration time of objects N1 and N2 both decreased in TLE 1 d and exhibited unobvious variation form 1 d to 14 d; however, there was no significant difference in the exploration time and discrimination ratio between object N1 and N2, no matter in NC or TLE groups. During the test phase, the rats preferred the new object N2’. Although the exploration time of both objects decreased after the onset of epilepsy, which was similar to the trend in the familiar phase, the exploration time in each trial of the new object N2’ was always greater than that of the old object N1. In the latent period (1 d to 5 d) of the TLE model, the discrimination ratio for the 2 objects was close to that of the NC group, whereas the discrimination ratio of N2’ significantly reduced in 14 d, which suggested the rats’ weakened preference for the novel object. The results revealed that the rats’ spatial cognition behavior was impaired by the spontaneous seizures during the chronic phase, whereas the impact was not evident during the latent period.

**Fig. 6.**
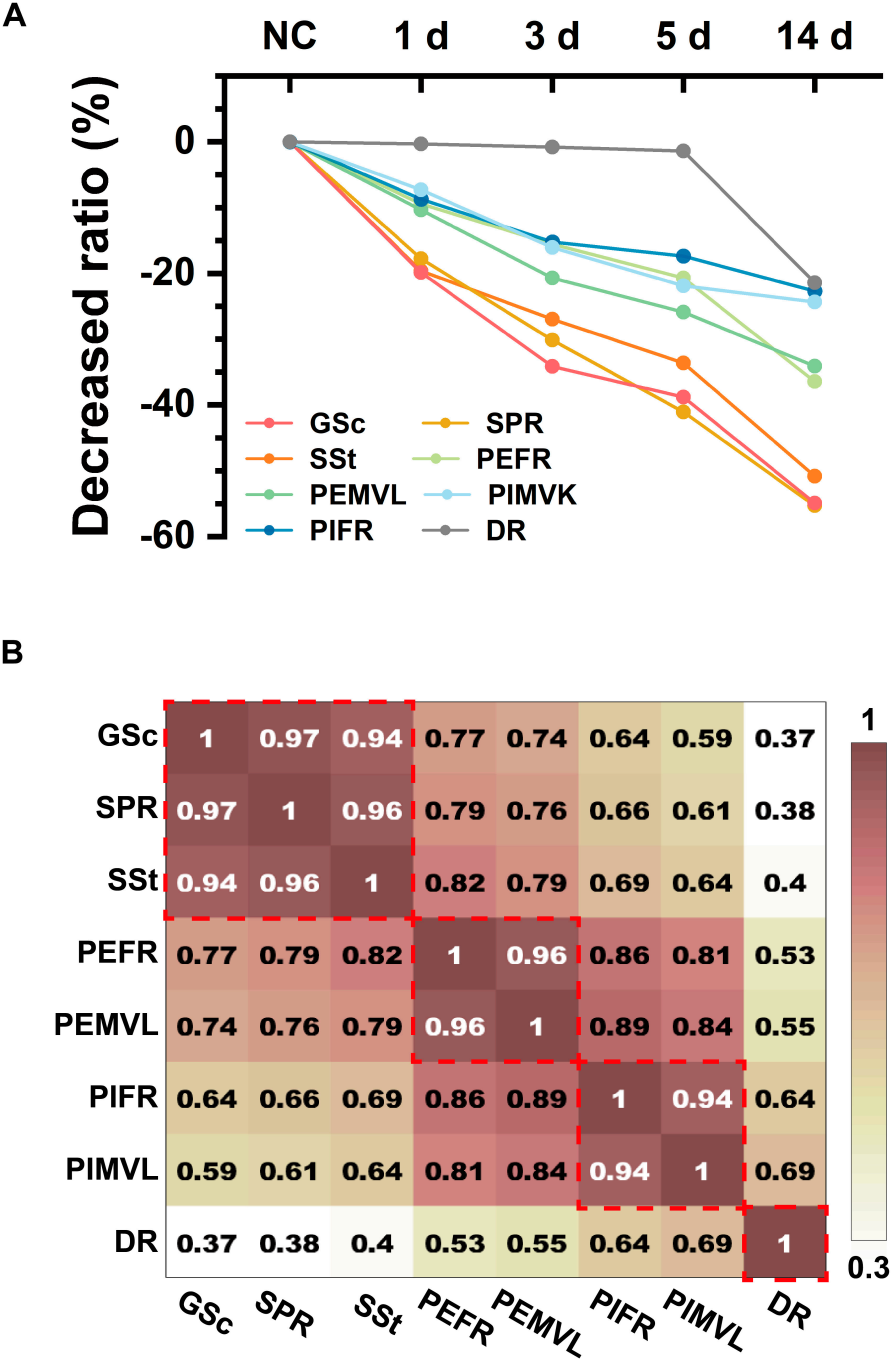
The decreased ratio of parameters from 3 aspects: representations of spatial cells, neurophysiology of MEC neurons, and behavior in the novel object recognition experiment. (A) Percentage changes with TLE duration of grid score (GSc), spatial peak firing rate (SPR), spatial stability (SSt), mean firing rate of PE (PEFR), mean vector length of PE (PEMVL), mean firing rate of PI (PIFR), mean vector length of PI (PIMVL), and discrimination ratio (DR). (B) Correlation matrix of the descending slope of GSc, SPR, SSt, PEFR, PEMVL, PIFR, PIMVL, and DR. The red rectangles indicate connected regions with high correlations.

As previously mentioned, our investigation into the effects of epilepsy included 3 aspects: (a) spatial representations of spatial cells, (b) neurophysiology of MEC neurons, and (c) behavior in the novel object recognition experiment. The rate of decline appeared to vary across these aspects. To examine this, we selected several parameters including grid scores (GSc) of grid cells, spatial peak firing rates (SPR) and spatial stabilities (SSt) of all spatial cells (grid, border, and NGB spatial cells), as well as the firing rate (PEFR) and phase locking strength (PEMVL) of PE neurons, firing rate (PIFR) and phase locking strength (PIMVL) of PI neurons, and discrimination ratios (DR) in the novel object recognition experiment. We calculated the percentage changes of these parameters in TLE rats compared to NC. As shown in Fig. 6A, GSc, SPR, and SSt exhibited the most rapid decline in the latent period and the greatest reduction at 14 d. PEFR and PEMVL showed a slower decline compared to the spatial representations but a larger decrease than PIFR and PIMVL. Furthermore, DR demonstrated minimal change during the latent period but exhibited a noticeable decrease in the chronic period. In conclusion, TLE had the most rapid and pronounced impact on the spatial cognitive cells, followed by gradual effects on the firing activities of neurons and their coupling with LFPs, ultimately influencing the behavior of rats. Finally, we calculated the correlations between the rate of decline of any 2 parameters, as shown in Fig. 6B. Our analysis revealed a correlation matrix with 4 distinct connected regions displaying high correlations, each corresponding to one of the 4 aspects: spatial representations (GSc, SPR, SSt), neurophysiology including PE (PEFR, PEMVL) and PI (PIFR, PIMVL), and behavior in the novel object recognition experiment (DR). These findings suggest a similar decreasing trend among parameters within their respective aspects.

## Discussion

In this study, we used the self-designed MEA to detect the firing activities of MEC neurons in the TLE rat models. The key findings of our research can be summarized as follows: (a) The spatial firing selectivity and temporal coding stability of MEC spatial cells containing grid, border, and NGB spatial cells were damaged in coincidence with duration of TLE. (b) After TLE modeling, the PE neurons and PI neurons in MEC gradually decreased their firing rate, with a more pronounced decline observed in the former. The theta oscillation of LFPs also weakened followed by the decreased theta power and theta phase locking in both types of neurons. (c) The recognition performance of epileptic rats in the novel object recognition experiment did not show a significant reduction in the latent period, despite the already evident impairment of spatial representations and firing properties of MEC neurons. Overall, our findings provide a perspective on how epilepsy affected the cognitive function.

The impairment of spatial cognitive function in TLE suggests the damage to the spatial information processing neural network. After pilocarpine treatment, grid, border, and NGB spatial cells exhibited a rapid decline in their spatial tuning. This decline may contribute to the observed reduction in spatial coding within the hippocampal place cells of epileptic rodents [17], which induced the impaired cognitive function. Consistent with previous research [18], our recordings also demonstrated that over 90% of the MEC neurons were spatial cells. Unlike grid cells and border cells, NGB spatial cells did not exhibit a specific firing pattern characterized by regularity in the number or positions of firing fields. However, our study revealed that NGB spatial cells responded differently to epilepsy compared to grid cells, suggesting a distinct spatial processing mechanism separate from grid cells. Grid cells maintained their modular organization with gradual changes in grid spacing and field sizes along the dorsal to ventral area of the MEC, with no apparent association with epilepsy. This finding suggests that the reduction in grid score was not due to distortions in the grid structure, but likely resulted from decreased spatial-specific firing and increased non-spatial-specific firing, as supported by the decrease in peak firing rate and the increase in sparsity. The modular organization of grid cells can serve as a metric for spatial information processing downstream in the hippocampus, which remained unaffected by short-term epilepsy in our study, confirming its ontogenetic stability.

MEC is the key brain region of seizure propagation in TLE. Dysfunction within its intrinsic circuit can result in synchronous hyperexcitability in the entorhinal–hippocampal network. Neurodegeneration in the MEC has been observed in TLE models through imaging and microscopic examination [19], consistent with the decreased firing rates of MEC neurons observed in our study. The greater decline in PE compared to PI indicated that excitatory neurons were more susceptible to the damage induced by epilepsy, which aligns with the histochemical report indicating greater loss of pyramidal cells than interneurons in TLE [20]. Although few studies have investigated theta oscillations in the MEC of TLE models, previous reports have demonstrated a progressive reduction in theta power of LFPs in the hippocampus with epilepsy duration [21,22]. One hypothesis is that the decrease in theta power is attributed to neuronal loss in the entorhinal cortex [23], which is supported by our findings. Theta oscillations are crucial for the spatial cognitive network, where excitatory neurons process spatial information through phase locking [24]. In addition, interneurons were believed to provide inhibitory connections for the network structure of grid cells, thus exhibiting the synchronous theta resonance [25]. Therefore, in open field tests, theta oscillations as well as the theta phase locking of both excitatory and inhibitory neurons showed the similar tendency with epilepsy, further verifying that inhibitory neurons were involved in the internal connections of grid cell networks. The restoration of the theta resonance network through interventions such as electrical stimulation or light stimulation of the entorhinal cortex may provide targeted treatment options for cognitive impairment induced by epilepsy.

We used the novel object recognition experiments to assess the cognitive function of epileptic rats. Unlike other commonly behavioral tests like Morris water maze [26] and Y maze [27], the test capitalizes on the rodents' natural inclination to explore novel stimuli, thereby minimizing stress and the likelihood of unexpected spontaneous seizures. In our experiment, rats with high anxiety due to epilepsy spent less time exploring both objects, consistent with the previous study [28]. However, epileptic rats still demonstrated a natural preference for exploring the novel object compared to the familiar one, indicating that their ability to recognize new objects remained intact during the latent period. However, at this time, the firing characteristics of neurons in MEC and the representations of spatial cells had been weakened. In the chronic period, the discrimination ratio was significantly lower, suggesting that the impairment of spatial learning and memory in the rats did not occur until the repeated spontaneous seizures. Our studies proved that there may be no substantial reduction in performance for simple spatial tasks during the latent period, even when the intrinsic circuitry of the MEC has already been compromised. Previous studies have demonstrated the importance of microelectrodes for early disease surveillance through biomarker detection [29–31]. Therefore, electrophysiological recordings utilizing MEA to analyze the spatial representations and firing characteristics of MEC neurons offer a promising approach for predicting TLE cognitive disorders associated.

In summary, we performed the self-designed MEA to detect neural activities in MEC of the TLE rat model. Following the lithium–pilocarpine treatment, we observed a decline in the spatial firing selectivity and stabilities of grid, border, and NGB spatial cells. Subsequently, we observed a decrease in the firing rate and theta locking strength of both excitatory and inhibitory neurons, accompanied by a reduction in theta oscillations in LFPs. Notably, the damage to excitatory neurons was more pronounced compared to inhibitory neurons. Ultimately, the rats’ spatial recognition ability declined, following the severe impairment of MEC neurons. Our study supported that the spatial representation and firing characteristics of MEC neurons could serve as potential “functional biomarkers” for predicting cognitive impairment in epilepsy.

## Materials and Methods

### Fabrication and modification of MEA

In our study, the 4-shank (32-channel) MEA was fabricated by micro-electromechanical system technology (Fig. S1) to monitor the neural electrophysiological signal of targeted area, especially for the MEC region. The step-arranged probes were according to the shape of MEC (Figs. S2A and B and S3A). After fabrication, the platinum nanoparticles were modified on the surface of the recording sites to enhance the specific surface area and improve the electrode conductivity (Fig. S2C and D). The detailed experimental process of MEA fabrication and modification was described in the previous report [32].

### Subjects

For the current study, 10 male Sprague-Dawley (SD) rats were used and divided equally into an epileptic group and a control group. We performed a power analysis to ensure the adequacy of the sample size (refer to the Supplementary Materials for details, Tables S1 and S2). All rats were implanted with MEA into the MEC region (anteroposterior: −8.9 mm, mediolateral: 4.5 mm, dorsoventral: 2 to 4 mm) by craniotomy when they weighed ~300 g. After 1 week of recovery and 3 d of behavioral testing, the lithium–pilocarpine treatment was performed for the epileptic group and saline treatment was performed for the control group at the same time. Each rat was housed individually at constant temperature (22 ± 1 °C) with a 12 h on/off light cycle, and the behavioral testing was carried out in the dark phase. All experiments were carried out with the permission of Beijing Association on Laboratory Animal Care and approved by the Institutional Animal Care and Use Committee at Aerospace Information Research Institute, Chinese Academy of Science (AIRCAS).

### Epileptogenesis

Previous studies have reported that the lithium–pilocarpine-induced epileptic rat model was similar to the clinical TLE [33]. This model can induce continuous generalized clonus by activating acetylcholine muscarinic receptors, resulting in epileptic seizures. The lithium–pilocarpine process was shown in Fig. S3B. First, rats were intraperitoneally (i.p.) injected with LiCl (127 mg/kg) to improve their sensitivities to the pilocarpine. After approximately 18 h, scopolamine methyl bromide (1 mg/kg) was injected (i.p.) into rats to alleviate peripheral cholinergic damage. Subsequently, rats received an injection (i.p.) of pilocarpine (10 mg/kg) 30 min later. In cases where no grand mal seizure occurred after the initial pilocarpine injection, a supplemental dose of 10 mg/kg was administered every 30 min until a seizure scored above fourth level on the Racine scales [34] was induced. The control rats were injected (i.p.) with the same amount of normal saline. The successful establishment of the epileptic rat models was determined by the occurrence of a grand mal seizure lasting over 1 h (Movie S1). After epileptogenesis, the behaviors and electrophysiological signal of rats were recorded for 2 h each day to detect the spontaneous epileptic activity (Fig. S9 and Movie S2). The duration of latency between the pilocarpine treatment and the first spontaneous seizure was 6 to 10 d. The experimental protocol including the open field test and the novel object recognition test was carried out in the latent period (1 to 5 d) and the chronic period (14 d).

### The open field test

The open field box was 120 cm × 120 cm × 60 cm in size made of gray acrylic plates. A white paper clue was placed in one wall of the box in order to facilitate the exploratory behavior of rats. During the experiment, the tested rats ran freely in the open field for 20 min, and the synchronous data containing neural signals and behavioral trajectories of rats were acquired (Fig. S3C).

The MEC neural signals at 30 kHz were recorded by the multilevel regulation and high-throughput neural signal detection instrument (AIRCAS-128, China) [35]. The LFPs were separated by a low-pass filter (0 to 250 Hz), and neural spikes were extracted from the high-frequency signals (>250 Hz) with the threshold about 3 times the baseline. In addition, the positions of running rats were acquired by the analysis software (EthoVision XT 15, Noldus, China) from the video recorded synchronously with neurophysiological data.

Before the formal exploration experiment, each rat underwent a familiarization period in the arena for a minimum of 30 min daily at the same time, over the course of 1 week. This procedure ensured that the rats became accustomed to the experimental environment. Subsequently, MEA was surgically implanted into MEC of the rats. Following a 1-week recovery, the aforementioned lithium–pilocarpine process was performed, and the behavioral experimental protocol was repeated in 1 d, 3 d, 5 d, and 14 d after.

### The novel object recognition test

The novel object recognition experiment consists of the following 2 phases: (a) the familiar phase, in which 2 objects (N1 and N2) were placed in the white cylindrical box (diameter: 80 cm) in advance. As illustrated in Fig. S3D, both objects shared the same color (yellow) and shape (cube: 10 cm × 10 cm × 10 cm) but were positioned differently. The rats were placed in the open field and allowed to freely explore for a duration of 5 min. (b) the test phase, where object N2 was replaced with a new object N2’ (cylinder: 8 cm diameter × 10 cm height), while object N1 remained unchanged. The rats were reintroduced to the arena for another 5 min of free exploration. The interval between 2 phases was 30 min. To minimize odor cues, the box and objects were wiped down with a 75% alcohol wipe between trials.

The exploration time (TN) for each object was defined as the duration that rats’ noses were within 5 cm of the object. To evaluate the preference for each object, we calculated the discrimination ratio (DR) for N2 using the following formula:

$$\mathrm{DR}=\frac{\mathrm{TN}2}{\mathrm{TN}1+\mathrm{TN}2}\times 100\%$$

The experimental protocol was performed at least 1 h after the open field experiment.

### Spike sorting

According to previous reports, the MEC units can be divided into 2 categories: PE neurons and PI neurons, distinguished by the firing rates and spike width [36]. Spike width of each unit was defined as the average value of spike waveform lengths from trough to the peak. All units were classified by *k*-means clustering into 2 types, in which the group with the lower mean firing rates and greater spike width was PE, while the other group was PI. Previous studies [18,37] have reported that many of PE in MEC exhibited stable spatial representations. Through the open field test, grid cells, border cells, NGB spatial cells, and nonspatial cells can be identified from recorded PE neurons (refer to the Supplementary Materials for details).

### Theta modulation analysis

The theta modulation encompasses 2 main aspects: the theta oscillation of LFPs and theta phase locking between spikes and theta rhythms. The theta ratio was calculated to evaluate the proportion of the theta band (4 to 8 Hz) in the frequency space (1 to 30 Hz) of PSD for both spikes and LFPs.

To examine the theta phase locking strength, the raw LFPs were band-pass filtered from 4 to 8 Hz, and the instantaneous theta phases φ_k_ were obtained by Hilbert transform. Then, the theta phase locking strength [7] was assessed by mean vector length (MVL) defined as:

$$\mathrm{MVL}=\left|\frac{1}{n}\sum_{k=1}^{n}e^{i\varphi_{k}}\right|$$

If spikes are locked at a specific phase, the values of spike phases would show a unimodal distribution, resulting in a larger mean vector length.

## Data Availability

All data needed to support the conclusions in the paper are provided in the paper and the Supplementary Materials.
