# Supplementary material for: Impaired Spatial Firing Representations of Neurons in the Medial Entorhinal Cortex of the Epileptic Rat Using Microelectrode Arrays
Source: Research (Wash D C). 2023 Sep 15;6:0229. doi: 10.34133/research.0229 (PMC10503993; doi:10.34133/research.0229)
Supplement: Supplementary 1 — Figs. S1 to S9 Tables S1 to S3 Movies S1 and S2 [file research.0229.f1.zip › Supplementary Materials.docx]

**Title**

Impaired spatial firing representations of neurons in the medial entorhinal cortex of the epileptic rat using microelectrode arrays

**Short title**

Impaired spatial cell tuning induced by epilepsy

**Authors**

Zhaojie Xu^1,2^, Fan Mo^1,2^, Gucheng Yang^1,2^, Penghui Fan^1,2^, Botao Lu^1,2^, Wei Liang^1,2^, Fanli Kong^1,2^, Luyi Jing^1,2^, Wei Xu^1,2^, Juntao Liu^1,2^, Mixia Wang^1,2*^, Yirong Wu^1,2*^& Xinxia Cai^1,2*^

**Affiliations**

*State Key Laboratory of Transducer Technology, Aerospace Information Research Institute, Chinese Academy of Sciences, Beijing 100190, China*

*^2^ University of Chinese Academy of Sciences, Beijing 100049, China*

^*^Address correspondence to: Mixia Wang; wangmixia@mail.ie.ac.cn and Yirong Wu; wyr@mail.ie.ac.cn and Xinxia Cai; xxcai@mail.ie.ac.cn

**1 Materials and Methods**

**1.1 Spatial cells identification**

To determine whether recorded PE cells in the medial entorhinal cortex(MEC) represented spatial features, we used spatial information (SI) score, which measures the mutual information between the rats' locations and the firing response of the cells [1]. The SI was calculated as follows:

Initially, the position data was discretized into bins of 2 × 2 cm^2^. In each bin with position $\boldsymbol{x}$, the firing rate $\lambda\left( \boldsymbol{x} \right)$ was spatially smoothed as below:

$$\lambda\left( \boldsymbol{x} \right)= {fr\times\sum_{i=1}^{m} g(\frac{\boldsymbol{s}_{\boldsymbol{i}}-\boldsymbol{x}}{\sigma})}/{\sum_{j=1}^{n} g(\frac{\boldsymbol{p}_{\boldsymbol{j}}-\boldsymbol{x}}{\sigma})}$$

where $g$ is a Gaussian ($e^{-x^{2}/2}$) kernel, $fr$ is the frame rate, $m$ is the number of spikes, $\boldsymbol{s}_{\boldsymbol{i}}$ is the position of the $i$-th spike, $\sigma$ is a smoothing coefficient, $n$ is the number of locations, $\boldsymbol{p}_{\boldsymbol{j}}$ is the position of the $j$-th frame. And the firing rate map was defined as the matrix $\Lambda\left( \boldsymbol{X} \right)$ containing the averaged firing rate $\lambda\left( \boldsymbol{x} \right)$ of all bins.

The spatial information (SI) was then given by:

$$SI=\sum\frac{\boldsymbol{\lambda}\left( \boldsymbol{x} \right)}{Ave(\boldsymbol{\lambda}\left( \boldsymbol{x} \right))}\boldsymbol{log}_{\mathbf{2}} \left( \frac{\boldsymbol{\lambda}\left( \boldsymbol{x} \right)}{Ave(\boldsymbol{\lambda}\left( \boldsymbol{x} \right))} \right)$$

During a 20-minute recording session of a single cell, spatial stability was defined as the correlation between the spatial firing rate matrices obtained during the first 10 minutes and the last 10 minutes of the trial.

For each recorded PE cell, we shuffled spikes relative to their associated positions 100 times and mark it as the spatial cell when its SI and spatial stability were both above 95% of the shuffled data as shown in Figure 1A. Spatial cells that did not meet the criteria for grid cells or border cells were designated as non-grid-border (NGB) spatial cells.

**1.2 Grid cells identification**

Grid cells were identified by the established methods [2, 3]. To determine whether the spike assembles formed a hexagonal pattern, the spatial autocorrelogram for the a firing rate map $\Lambda\left( \boldsymbol{X} \right)$ was calculated, where each element $\gamma(\boldsymbol{\tau})$ with $\boldsymbol{\tau}$ offset relative to the origin was estimated as:

$$\gamma(\boldsymbol{\tau})=r(\Lambda\left( \boldsymbol{X} \right) , \Lambda\left( \boldsymbol{X}-\boldsymbol{\tau} \right))$$

where $r$ is the Pearson’s correlation coefficient:

$$r\left( \mathbf{A},\boldsymbol{B} \right)=\frac{\sum\sum\left( \boldsymbol{A}-\bar{\boldsymbol{A}} \right)\left( \boldsymbol{B}-\bar{\boldsymbol{B}} \right)}{\sqrt{(\sum\sum\left( \boldsymbol{A}-\bar{\boldsymbol{A}} \right)^{2})(\sum\sum\left( \boldsymbol{B}-\bar{\boldsymbol{B}} \right)^{2})}}$$

To quantify the gridness of the autocorrelogram, we assessed its rotational symmetry $\rho(\varphi)$ by correlating the autocorrelogram rotated at angle $\varphi$ with the original. The circular region encompassing the innermost six peaks, excluding the center, represented the area of correlation. The grid score was then defined as：

$$grid score=min\left( \rho\left( 60^{\circ} \right)，\rho\left( 120^{\circ} \right) \right)-max\left( \rho\left( 30^{\circ} \right)，\rho\left( 90^{\circ} \right)，\rho\left( 150^{\circ} \right) \right)$$

For each trial, we used 100 random shuffling of spike locations for each trial. A grid cell was identified when its grid score exceeded the 95th percentile of grid scores obtained from the shuffled data, as shown in Figure S5A.

The grid spacing was calculated as the average distance between the centers of adjacent firing fields observed in the autocorrelogram.

**1.3 Border cells identification**

Border cells were characterized by firing fields arranged along the boundary [4]. They were identified as follows: Firstly, for the firing rate map of each PE neuron, the edge threshold was set at 0.2 times the peak firing rate. Potential border firing fields were identified as contiguous regions with firing rate above the threshold and covering an area of at least 200 cm².

Subsequently, the areas of each potential border firing fields were calculated as $c_{m}$, and the average distance of each pixel in the field to the nearest wall was $d_{m}$ . Then the border score was defined as：

$$border socre=\frac{{c_{m}-d}_{m}}{{c_{m}+d}_{m}}$$

Finally, each border cell was identified when its border score was greater than the 5th largest one of the 100 shuffled data as shown in Figure S5B.

**1.4 Spatial cells analysis**

As for each spatial cell, the peak firing rate was defined as the maximum of the firing rate map $\boldsymbol{\Lambda}\left( \boldsymbol{X} \right)$.

The sparsity index [5] was defined as:

$$Sparsity=\frac{\left( \sum\boldsymbol{\lambda}\left( \boldsymbol{x} \right) \right)^{\boldsymbol{2}}}{{\sum\boldsymbol{\lambda}\left( \boldsymbol{x} \right)}^{\boldsymbol{2}}}$$

For each firing rate map, the spatial firing fields were identified as connected regions with an area greater than 30 cm^2^ where$\lambda\left( \boldsymbol{x} \right)$ exceeds 20% of the peak firing rate.

The field size of a spatial cell was calculated by the equivalent diameter of all firing fields.

**1.5 Power analysis**

The sample size in the TLE group was counted by the number of detected cells from 5 TLE rats in each trial as shown in Table S1. We applied the student t-test to reveal the significant difference in the Fig. 1-Fig. 5B. Based on this, power analysis was performed to assess whether the sample size (SZ) had sufficient statistical power. Sample size formula [6] is as below:

$SZ=2\left[ \frac{\left( Z_{\alpha}+Z_{\beta} \right)\sigma}{\delta} \right]^{2}$,

where $\delta$ is the effect size defined as the difference between the two means and $\sigma$ is the pooled standard derivation. $Z_{\alpha/2}$ and $Z_{\beta}$ are taken from the standard normal distribution $Z_{\alpha}$ = 1.65 (Type I error 0.05) and $Z_{\beta}$ = 1.28 (Type II error 0.1; power 90%).

The minimum sample size in the TLE group calculated by the power analysis was shown in Table S2.

**1.6 Analysis of variance**

We computed the mean sparsity, mean peak firing rate, mean NGB field size, and mean spatial stability across all cells within each of the ten rats. To investigate the distinctions between the NS group and the TLE group at each of the five measurement instances, we utilized an ANOVA model implemented with SPSS software. The results of these analyses are shown in Table S3.

**2 Supporting figures**

***
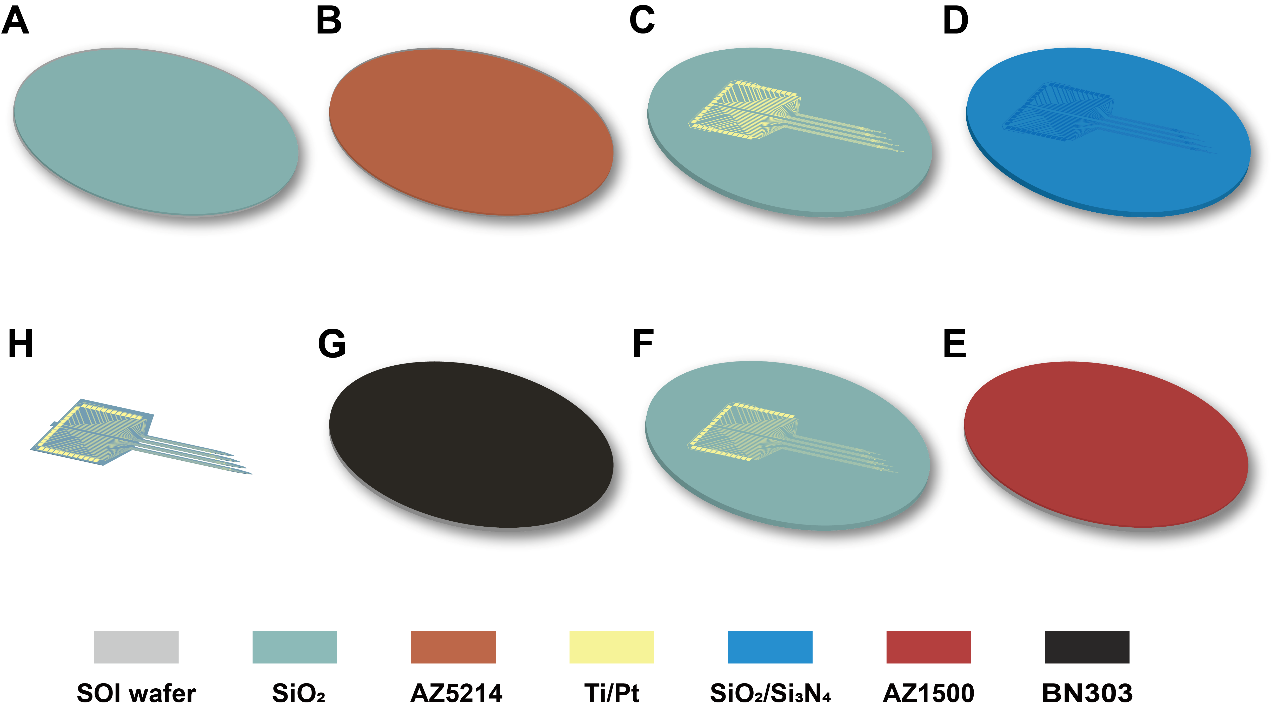
***

**Fig. S1.** **Schematic illustration of MEA fabrication process.** (A) Thermal oxidation of SOI wafer to produce the SiO_2_ film. (B) AZ5214 photoresist was spin‐coated to photoetch the metal layer. (C) Deposition of Ti/Pt and lift-off to form the conductive layer. (D) Deposition of Si3N4/SiO2 to establish the insulator layer. (E) AZ1500 photoresist was spin‐coated to photoetch the insulator area. (F) Selectively deep etching to define the shape of MEA. (G) Spin‐coating of BN303 photoresist to protect MEA during the back wet etching. (H) Release of MEA through wet etching.

***
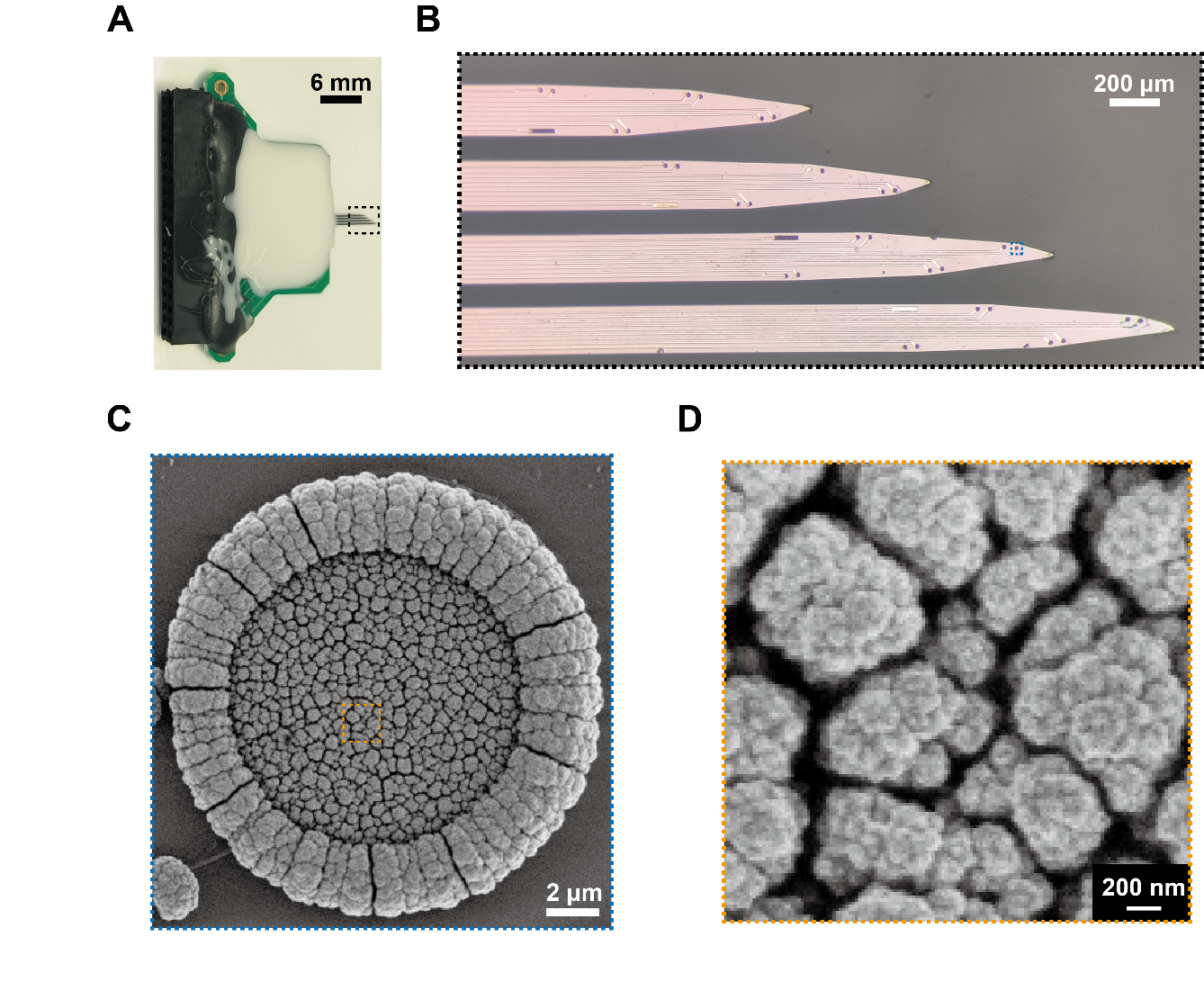
*Fig. S2. Optical photograph and morphology of the self-designed MEA and modified recording sites.** (A) The optical photograph of individual packaged MEA. (B) The microscopic image of the top of MEA probes. (C) SEM images of a single recording site after modification with PtNPs. (D) Local 10 × magnification display of C.

*
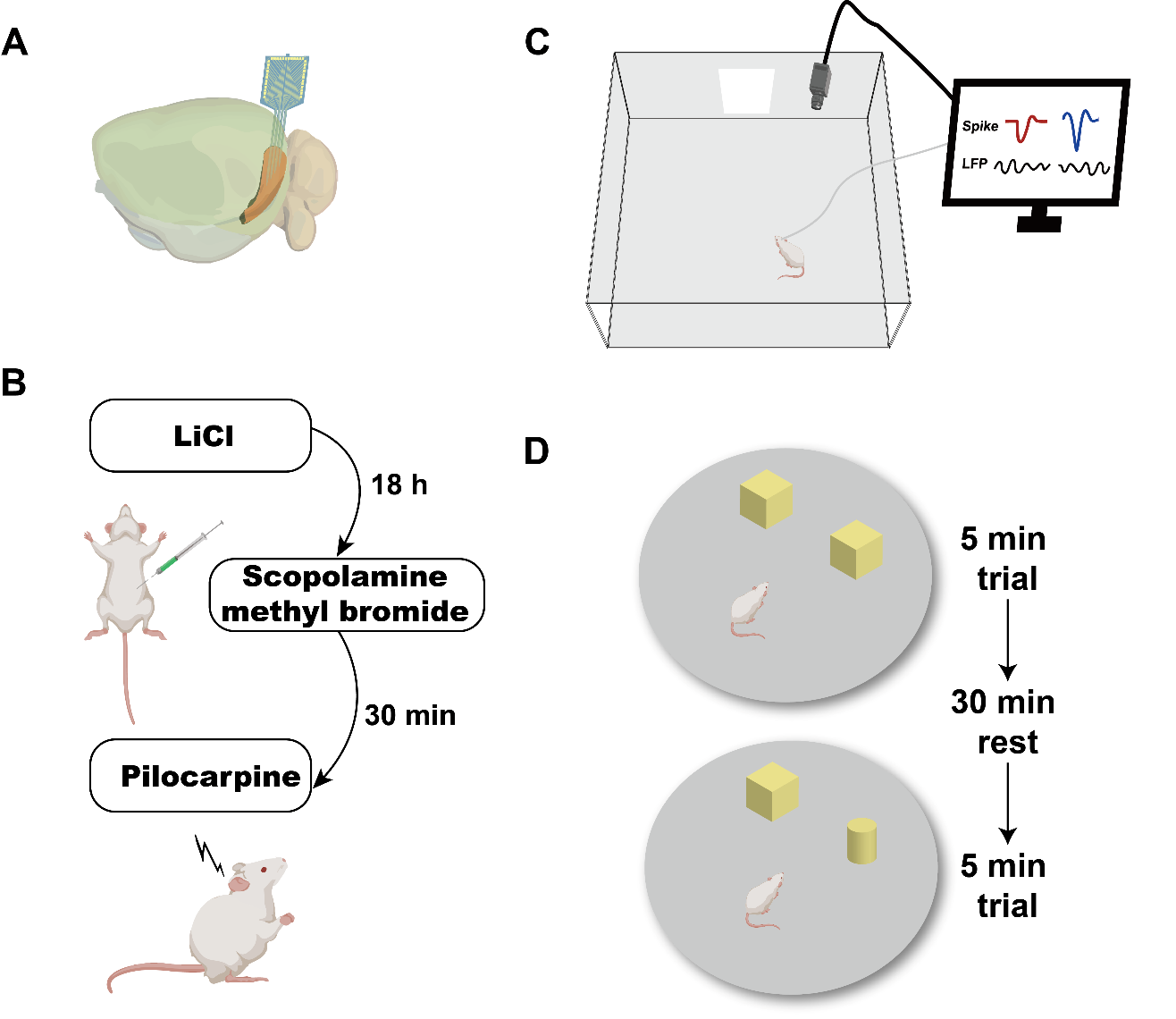
*

**Fig. S3. Illustration of experimental procedure.** (A) MEA was designed to implant into MEC region. (B) Schematic illustration of lithium-pilocarpine induced epileptic rat model. (C) The open field test recording the rat’s trajectories and spikes synchronously. (D) Schematic illustration of the novel object recognition test.


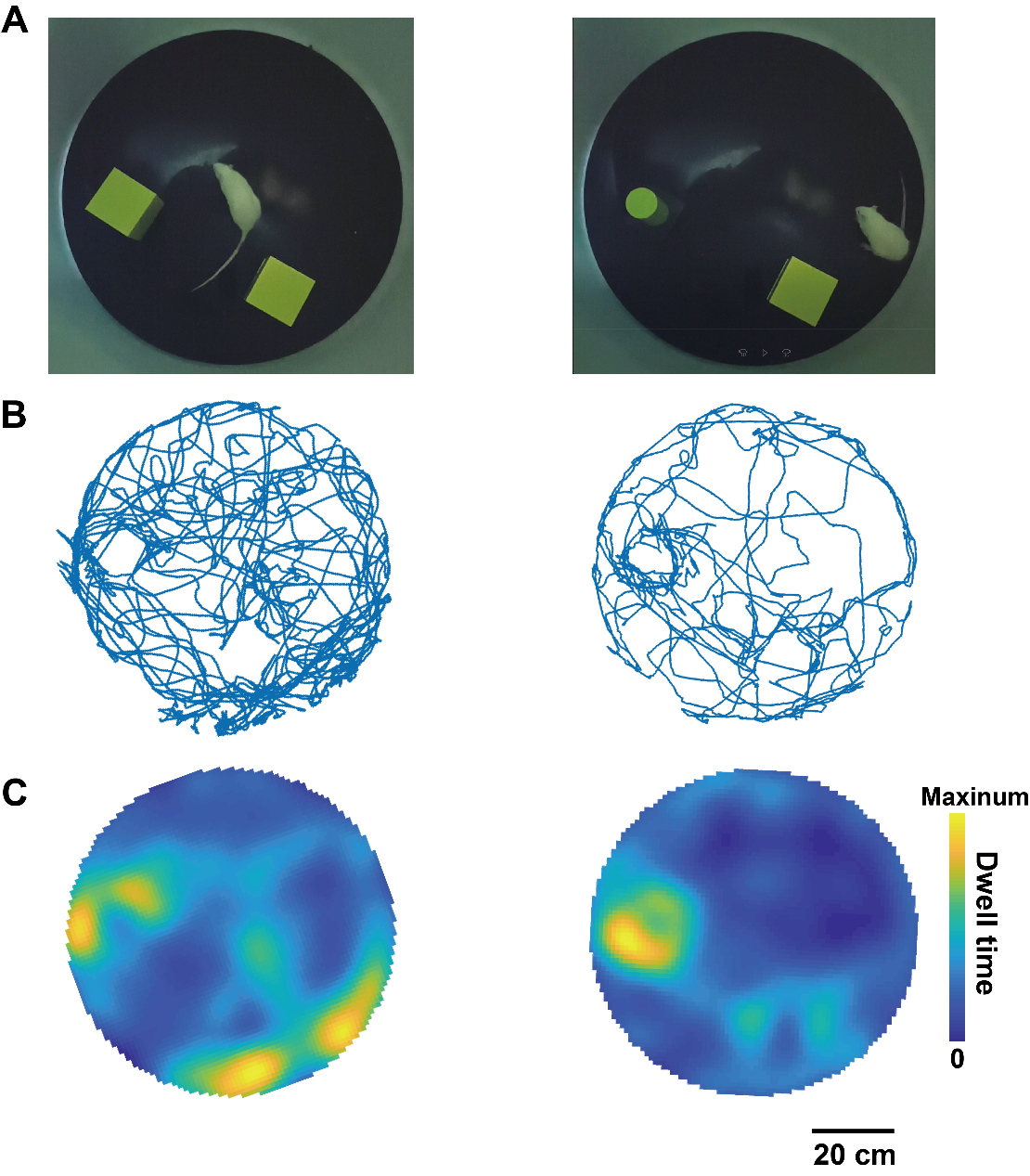


**Fig. S4. Performance of the tested rat in the novel object recognition test.** (A) Snapshots of a recording video in the familiar phase (left) and test phase(right). (B) Trajectories of the tested rat in the familiar phase (left) and test phase(right) according to A. (C) Heat maps showing the dwell time of all positions in the arena according to B.


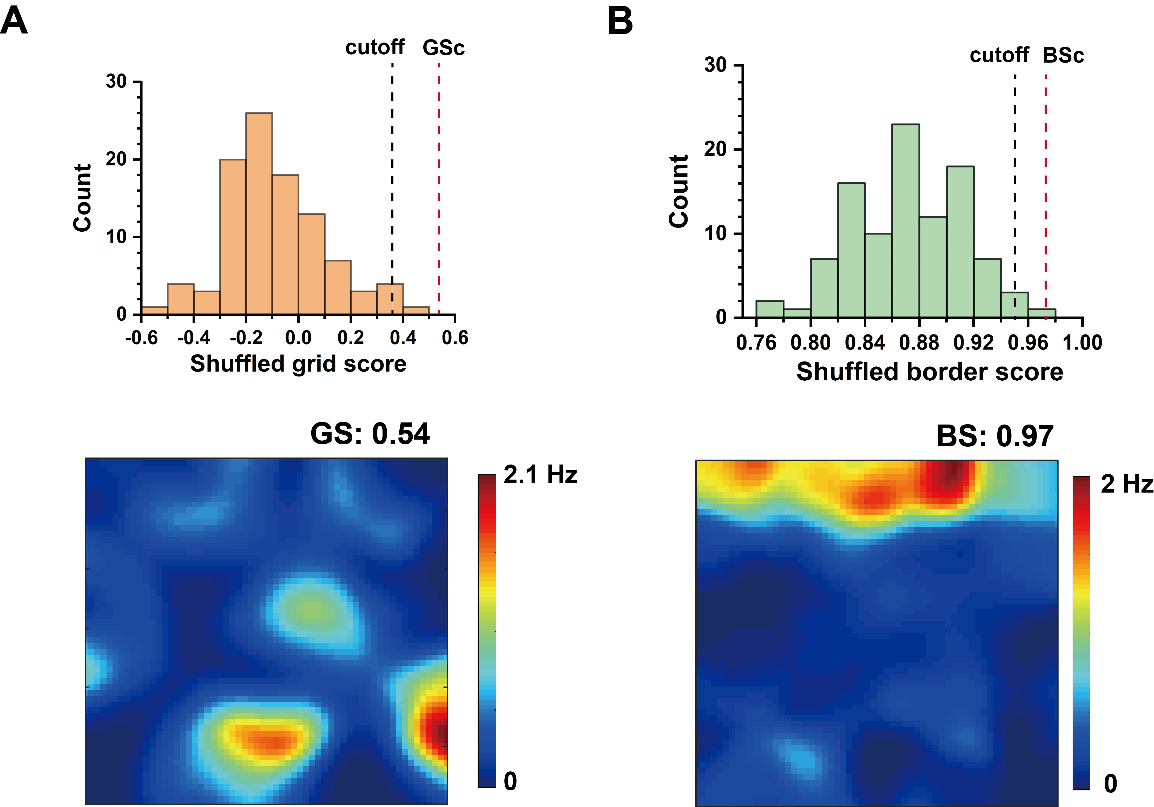


**Fig. S5.** **Identification of the grid cell and border cell by 100 shuffled data.** (A) The distribution of grid scores from 100 shuffled data (top) of the grid cell spike firing shown in the firing rate map (bottom). The red line indicates the real grid score (GSc) of the grid cell and the black line indicates the 5th largest grid score of 100 shuffled data. (B) The distribution of border scores from 100 shuffled data (top) of the border cell spike firing shown in the firing rate map (bottom). The red line indicates the real border score (BSc) of the grid cell and the black line indicates the 5th largest border score of 100 shuffled data.


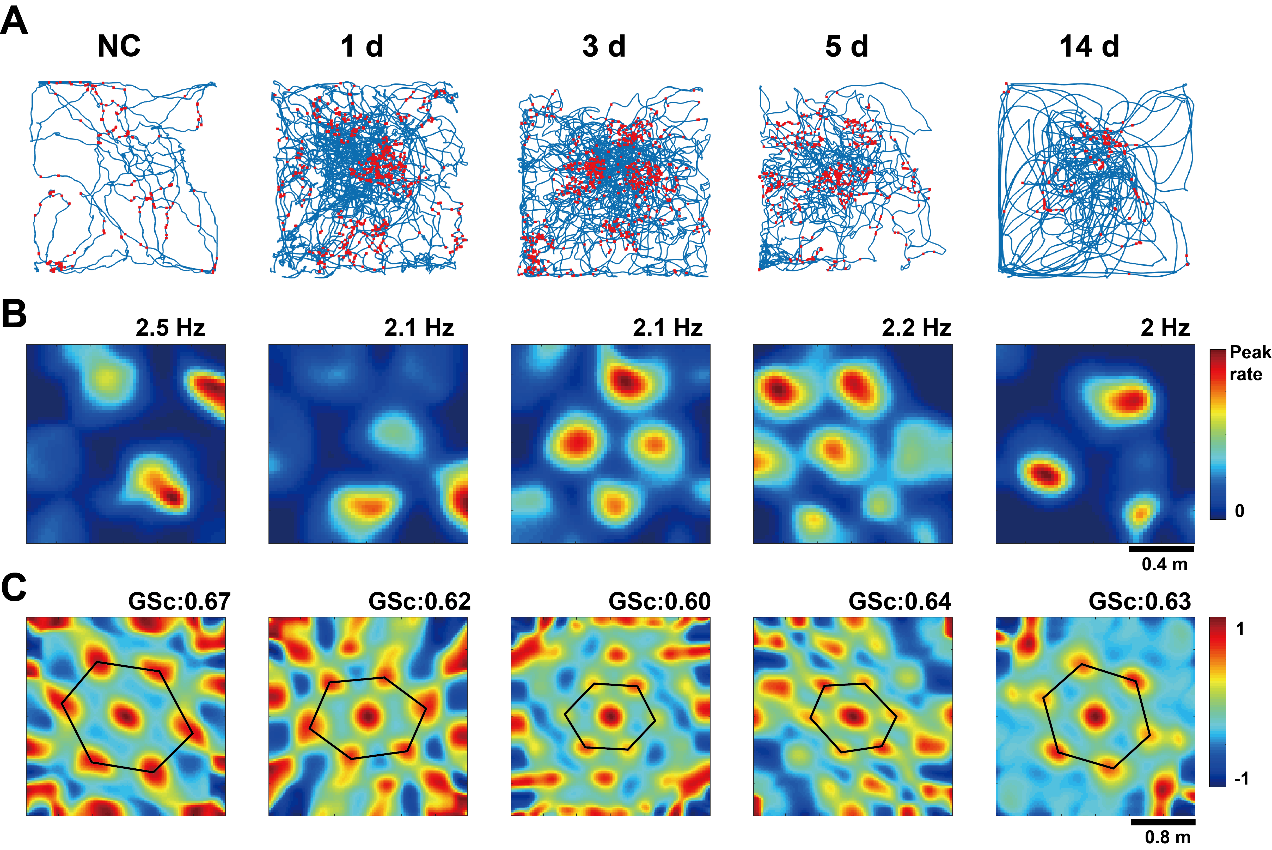


**Fig. S6. The typical grid cells from NC to 14d in the NS group.** (A) The trajectory maps with red spike position dots of grid cells from NC to NS 14d. (B) The spike firing rate maps according to A with the peak firing rates on the top. (C) The autocorrelogram of spike firing rate maps in B with the grid scores on the top. The black lines indicated the hexagonal structure of firing fields for each cell.

**Fig. S7. The decline of spatial representations with the duration of TLE.** (A) The sparsity of all spatial cells gradually increases from NC to TLE 14d compared to NS (unpaired t-test, *p<0.05, ****p<0.0001). (B) The peak firing rates of all spatial cells gradually reduce from NC to TLE 14d compared to NS (unpaired t-test, *p<0.05, ****p<0.0001). (C) The field sizes of NGB spatial cells gradually increase from NC to TLE 14d compared to NS (unpaired t-test, *p<0.05, ****p<0.0001). (D). The spatial stabilities of all spatial cells gradually reduce from NC to TLE 14d compared to NS (unpaired t-test, *p<0.05, ****p<0.0001).
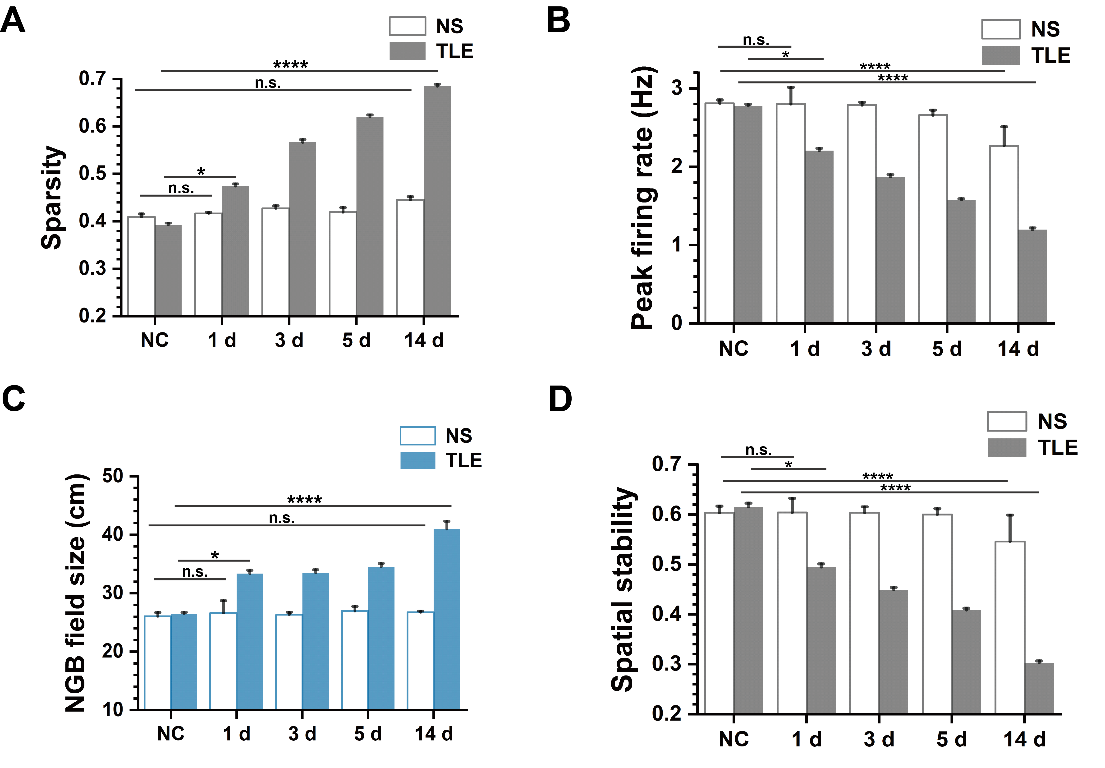


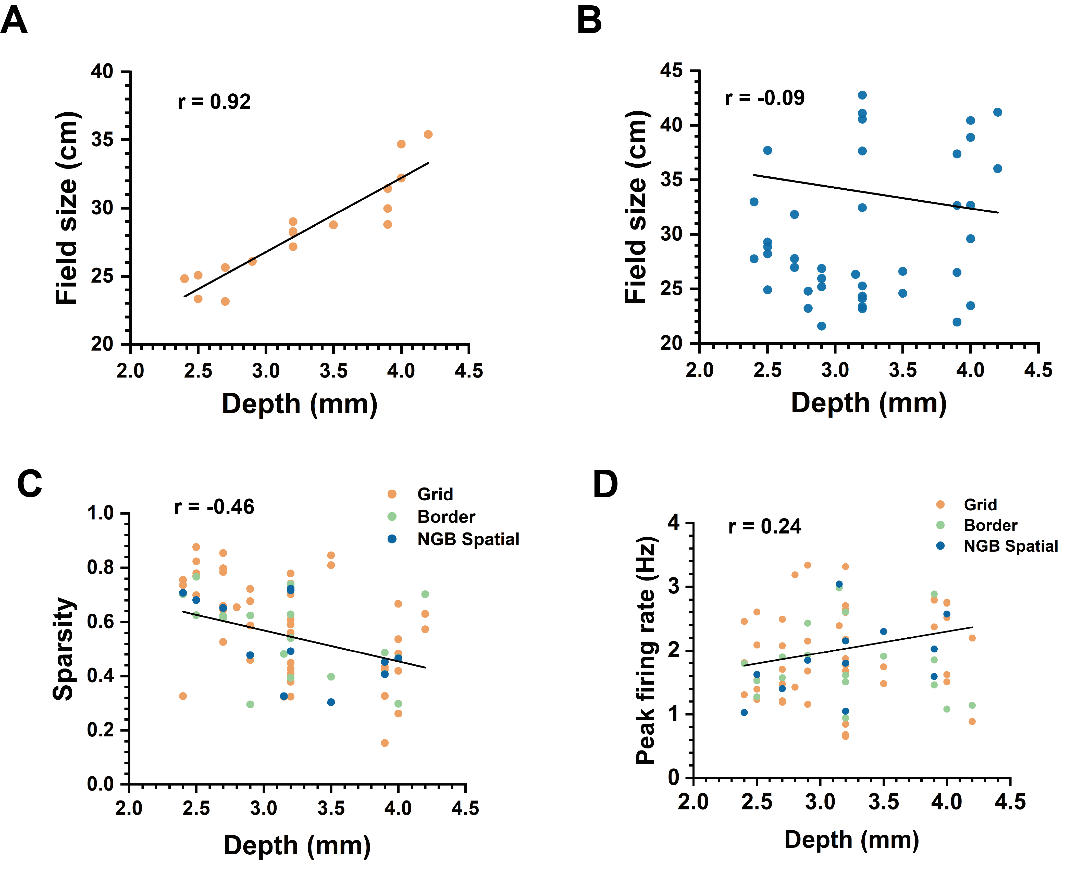


**Fig. S8. The relationship between the spatial representations and the depth of MEC spatial cells.** (A) The field sizes of grid cells exhibited a linear relationship to the depth. (B) The field sizes of NGB spatial cells exhibited a poor linear relationship to the depth. (C) The sparsity of grid, border and NGB spatial cells exhibited a poor negative linear relationship to the depth. (D) The spatial peak firing rates of grid, border and NGB spatial cells exhibited a poor positive linear relationship to the depth.


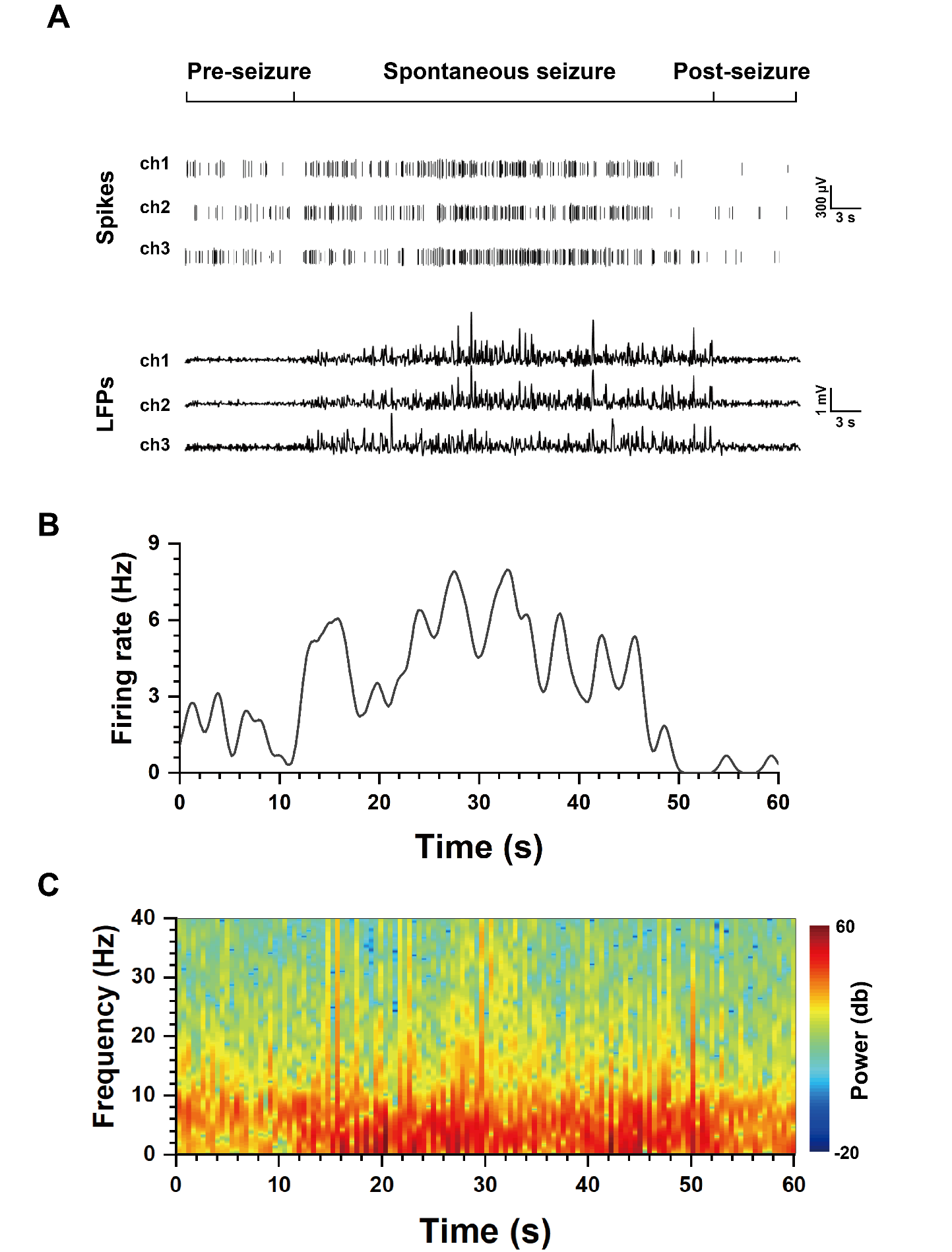


**Fig. S9. Dynamic variations of electrophysiology activities recorded in MEC throughout a typical spontaneous seizure.** (A) Three channels of neural spikes (top) and LFPs (bottom) recorded for 1 minute, including three periods of pre-seizure, spontaneous seizure and post-seizure**.** (B) The spike firing rate corresponding to ch1 in (A). (C) The short-time power spectral density of LFPs corresponding to ch1 in (A).

**Table S1. The actual sample sizes corresponding to Fig.1-Fig.5.**

|  | Grid cell | NGB cell | All spatial cell | PE | PI | Rat |
| --- | --- | --- | --- | --- | --- | --- |
|  | Fig.1E | Fig.2E | Fig.2C&D&F | Fig.3C&E, Fig.4F | Fig.3C&E, Fig.4F | Fig. 5B&C |
| NC | 4 | 12 | 19 | 22 | 5 | 5 |
| 1 d | 4 | 11 | 19 | 24 | 4 | 5 |
| 3 d | 3 | 14 | 23 | 26 | 3 | 5 |
| 5 d | 4 | 13 | 23 | 25 | 4 | 5 |
| 14 d | 3 | 13 | 22 | 24 | 4 | 5 |

**Table S2. The sample sizes calculated by the power analysis in Fig.1-Fig.5.**

|  | Grid cell | NGB cell | All spatial cell | PE | PI | Rat |
| --- | --- | --- | --- | --- | --- | --- |
|  | Fig.1E | Fig.2E | Fig.2C&D&F | Fig.3C&3E, Fig.4F | Fig.3C&E, Fig.4F | Fig. 5B&C |
| NC vs 1d | 3 | 12 | 18 | - | - | - |
| NC vs 3d | 2 | 11 | 10 | 16 | 2 | - |
| NC vs 5d | 2 | 10 | 8 | 13 | 2 | - |
| NC vs 14d | 1 | 5 | 2 | 8 | 2 | 1 |

**Table S3. The differences of spatial representations within intra-group and inter-group.**

|  | **sparsity** | | **peak firing rate** | | **NGB field size** | | **spatial stability** | | |
| --- | --- | --- | --- | --- | --- | --- | --- | --- | --- |
|  | F | Sig. | F | Sig. | F | Sig. | F | Sig. |  |
| NS group | 0.658 | 0.647 | 2.013 | 0.231 | 0.043 | 0.995 | 0.976 | 0.495 |  |
| TLE group | 134.876 | <0.001 | 10.309 | 0.012 | 30.083 | 0.001 | 22.579 | 0.002 |  |
| inter-group | 42.280 | <0.001 | 37.653 | <0.001 | 12.821 | 0.007 | 15.327 | 0.004 |  |

**References**

1. Savelli, F., Yoganarasimha, D., & Knierim, J. J. (2008). Influence of boundary removal on the spatial representations of the medial entorhinal cortex. *Hippocampus*, *18*(12), 1270–1282. https://doi.org/10.1002/hipo.20511

2. Sargolini, F. (2006). Conjunctive Representation of Position, Direction, and Velocity in Entorhinal Cortex. *S*cience, 312(5774), 758–762. https://doi.org/10.1126/science.1125572

3. Gerlei, K., Passlack, J., Hawes, I., Vandrey, B., Stevens, H., Papastathopoulos, I., & Nolan, M. F. (2020). Grid cells are modulated by local head direction. Nature Communications, 11(1), 4228. https://doi.org/10.1038/s41467-020-17500-1

4. Solstad, T., Boccara, C. N., Kropff, E., Moser, M.-B., & Moser, E. I. (2008). Representation of Geometric Borders in the Entorhinal Cortex. Science, 322(5909), 1865–1868. <https://doi.org/10.1126/science.1166466>

5. Sharif, F., Tayebi, B., Buzsáki, G., Royer, S., & Fernandez-Ruiz, A. (2021). Subcircuits of Deep and Superficial CA1 Place Cells Support Efficient Spatial Coding across Heterogeneous Environments. Neuron, 109(2), 363-376.e6. https://doi.org/10.1016/j.neuron.2020.10.034

6. Carlin, J. & Doyle, L. (2002). Sample size. Journal of Paediatrics and Child Health, 38, 300–304.
